# Enhanced degradation of anthraquinone dyes by microbial monoculture and developed consortium through the production of specific enzymes

**DOI:** 10.1038/s41598-021-87227-6

**Published:** 2021-04-07

**Authors:** Swati Sambita Mohanty, Arvind Kumar

**Affiliations:** Department of Chemical Engineering, National Institute of Technology Rourkela, Rourkela, Odisha 769008 India

**Keywords:** Biological techniques, Biotechnology, Environmental sciences

## Abstract

The current study investigates the decolorization of Indanthrene Blue RS dye and the optimization of process parameters needed for effective decolorization by the bacterial consortium. The pure culture of strain TS8, PMS, and NCH has been isolated from the textile wastewater sample collected from local textile processing units outlet and dye contaminated soil from Odisha, India. A bacterial consortium-BP of *Bacillus flexus* TS8 (BF), *Proteus mirabilis* PMS (PM), and *Pseudomonas aeruginosa* NCH (PA) were developed. The physicochemical parameters were optimized to attain maximum decolorization efficacy. Degradation of Indanthrene Blue RS and the formation of metabolites were confirmed through UV–vis spectroscopy, FT-IR, and GC–MS analysis. The developed consortium-BP showed an enhanced decolorization of Indanthrene Blue RS dye with an Average decolorization rate of 11,088 µg h^−1^ within 9 h compared to the individual strains under aerobic conditions. The supplementation of agricultural residual wastes showed increased decolorization efficiency of consortium-BP. Higher reduction in TOC and COD removal (≥ 80%) determined the mineralization of Indanthrene Blue RS by consortium-BP. Significant induction of various oxidoreductive enzymes in consortium-BP compared to that of Individual strains indicates their involvement in the overall decolorization and degradation process, with the higher protein concentration in the intracellular enzymes. Studies on the phytotoxicity effect revealed the non-toxic nature of the degraded products formed on mineralization of Indanthrene Blue RS by consortium-BP. This study represents a new approach for enhanced biodegradation using consortium-BP in treating textile wastewaters containing anthraquinone dyes.

## Introduction

Synthetic dyes such as azo and anthraquinone dyes are commonly used in food, paper, cosmetics, textiles, leather, and pharmaceutical industries because they are easy to synthesize, cost-effective, stable compared to natural colors, and are available in various colors^1^. Anthraquinone dyes are resistant to degradation and persist for an extended period in the environment because of their fused aromatic structure^2^. Textile dye effluents, containing suspended particles, detergents, and dyestuff when improperly disposed into water bodies, lead to reduced sunlight penetration and subsequently leading to decreased photosynthetic activity, reduced dissolved O_2,_ and deterioration in the quality of water. This water containing textile effluents has potential acute toxicity effects on aquatic life and contributes to ecological damages^3,4^. High total organic carbon (TOC) and chemical oxygen demand (COD) complicate the effluent treatment, resulting in potential ecological damage due to changes in pH, increased levels of COD, often causing the discoloration of the water resources into which they are released^5^. These anthraquinone dyes and their metabolites released in the effluents are both carcinogenic and mutagenic to life forms^6–8^ and pose potential risks to human health^9^.

Several conventional physicochemical methods, such as physical methods (i.e., coagulation or adsorption on activated carbon) and chemical methods (electrolysis, advanced oxidation, reverse osmosis, and ozonation process), were used in decolorizing textile wastewater^10^. These methods have several drawbacks, such as not being economically feasible, incomplete removal of the recalcitrant dyes and their metabolites, and increased sludge generation, causing secondary pollution, making them difficult to implement^11^. However, most textile effluents containing dyes cannot be treated by conventional processes and remain undegraded in the environment for a considerable length of time because they are stable against the light, temperature, and oxidizing agents. The main goal of this emerging microbial decolorization and degradation strategy is the remediation of dye contaminated wastewater. Compared to physicochemical approaches, the biological treatment method is more economical and generates less sludge and non-toxic metabolites^12^. The microorganism’s activity and adaptability determine the efficacy of microbial decolorization. This biodegradation process has turned out to be a promising method as it completely decolorizes the dye and transforms them into a non-toxic chemical form^13,14^. Under optimized aerobic or anaerobic conditions using microorganisms, a higher degree of decolorization and dyes degradation can be achieved^15–17^. A wide range of organisms capable of degrading textile dyes has been reported in the literature, with most of the studies focused on bacteria and fungi^18,19^.

The bioremediation of textile wastewaters is continuously expanding within environmental biotechnology as it is a fast and efficient technology for the continuous removal of colour. Pure bacterial strains generally are incapable of degrading the dyes completely, producing carcinogenic aromatic amines as intermediates, which further needs to be decomposed^20^. It is essential to scale up and maintain large scale pure cultures for wastewater treatment systems^21^. In the recent past, many consortiums having enhanced degradation abilities have been studied. They have demonstrated a significant level of mineralization and biodegradation by the microbial consortium in degrading the synthetic dyes than the individual pure strains because of the synergistic behavior in the metabolism of the microbial population^22^. The bacterial strains in the consortium metabolize the molecular structure of the dye by treating different aromatic ring positions, and the metabolites formed were further degraded by the surviving strains^10,23^. Several researchers are currently working on developing an efficient microbial consortium to enhance the decolorization efficiency that can remove a variety of pollutants from textile wastewater. Some researchers developed a bacterial consortium using *Bacillus cereus* (BN-7), *Pseudomonas putida* (BN-4), *Pseudomonas fluorescens* (BN-5), and *Stenotrophomonas acidaminiphila* (BN-3). They found that the developed consortium showed an increased decolorization efficiency of 78–99% with the dye concentration of 60 mg L^−1^ (acid red-88, acid red-119, acid red-97, and acid blue-113) within 24 h, suggesting three times higher decolorization efficiency than the individual strain^24^.

The decolorization kinetic of remazol black-B in a continuous anoxic–oxic reactor with a developed bacterial consortium of *Pseudomonas aeruginosa, Rhodobacter sphaeroides, Proteus mirabilis* NAD1, and NAD6 and *Bacillus circulans* was reported by Dafale et al.^25^. They observed that the consortium exhibited a color reduction of ≥ 90% and a COD reduction of 80% using synthetic wastewater with a dye concentration of 100 mg L^−1^ dye within 24 h of incubation. The developed consortium GG-BL consisting of *Galactomyces geotrichum* MTCC 1360 and *Brevibacillus laterosporus* NCIM 2298 for decolorization of golden yellow HER under optimized sequential aerobic and microaerophilic conditions was demonstrated by Waghmode et al.^26^. The findings showed that within 24 h of incubation, the consortium GG-BL could effectively reduce the color, COD, and TOC by 100%, 84%, and 63%, respectively. The metabolite formed on mineralization suggests the presence of decolorizing enzymes such as NADH-DCIP reductase, laccase, veratryl alcohol oxidase, tyrosinase, azo reductase, and riboflavin reductase enzyme degrading the dye into non-toxic compounds. Similarly, several results were documented for the efficient decolorization of 90% for reactive red-3BS, 70% for reactive red-195, and 93.7% for acid blue-113 by a specific microbial consortium under aerobic conditions^23,24^.

The decolorization of reactive green-19 dye using bacterial consortium comprising of *Zobellella taiwanensis* AT1–3, and *Bacillus pumilus* HKG212 under static condition was reported by Das and Mishra. With the addition of yeast extract as co-substrate, they observed a decolorization efficiency of 97% with an initial dye concentration of 100 mg L^−1^ within 24 h^27^. The available literature showed that the microbial consortium could effectively decolorize the reactive dyes in a shorter time relative to the individual microbial strain. During the decolorization process, it is difficult to determine the impact of experimental conditions. However, trials on bacterial-bacterial synergism are being used in developing new, environmentally friendly remediation technologies for the degradation of textile effluent without producing toxic metabolites. This emerging approach to microbial decolorization and degradation aims primarily at remediation of dye contaminated wastewater due to its cheaper and environment-friendly nature with less sludge and the production of non-toxic metabolites compared to physicochemical methods^12^.

Microorganisms can decolorize the dye with different enzymatic systems. Microbial decolorization of the dyes is associated with the enzymatic process by utilizing enzyme systems such as lignin peroxidase, laccase, tyrosinase, and azoreductase of the intracellular and extracellular oxidoreductase enzymes^28,29^. Under aerobic conditions, the microbes should be adapted and take a long growth phase in continuous operation. Thakur et al. reported that the bacteria metabolize the reductase enzyme in the cytoplasm, which is distinct to reactive dyes and reduces the chemical bond under optimal conditions^30^. The biodecolorization of acid black-24 by *Bacillus halodurans* MTCC 865 under aerobic static conditions exhibited ≥ 90% decolorization efficacy at pH 9 and 37 °C within 6 h treatment^31^. However, trials on bacterial-bacterial synergism are being used to develop new, environmentally friendly remediation technologies for textile effluent degradation producing none or minimal potentially toxic metabolites^32,33^. Enzymatic treatments have reduced environmental effects because they do not involve any risk of contamination. The enzymatic operation has been established in textile industries and has become extremely significant as biocatalysts. Biotechnology and environmental applications are focused on the identification of the microbial degradation pathway^34,35^.

In addition to the use of enzymes, bio-sorbents from agricultural waste were utilized for the elimination of dyes from wastewater in a cost-effective and eco-friendly manner. Several non-renewable natural sorbents such as rice husk, sugarcane bagasse, brown seaweed biomass, eucalyptus bark, fly ash, coal, peat, coconut husk, and chitosan were evaluated to minimize the processing cost and making it more viable for their dye removal efficiency^36,37^. Dong et al. isolated *Rhodocyclus gelatinosus* XL-1 under anaerobic conditions from contaminated wastewater. They studied the decolorization of reactive brilliant blue (KN-R) using isolated strain and found that ≥ 93% decolorization efficacy was achieved. They suggested that the decolorization of the dye resulted due to the use of peptone as a substrate. The microbe uses the dye as a co-substrate, and the decolorization efficiency has been improved by adding peptone where the degradation resulted through mineralization, hydrolysis, and co-metabolism process^38^.

Similarly, Xu et al. reported the decolorization of reactive brilliant blue K-GR under the anaerobic condition using *Shewanella decolorationis* S12 isolated from textile wastewater. They observed that 99% of decolorization was achieved through flocculation with the dye concentration of 50 mg L^−1^ within 15 h. The increase in the decolorization was due to the supplementation of various carbon sources such as peptone, lactate, succinate, formate, and yeast extract, whereas the decolorization efficiency decreased with HgCl_2_^39^.

The present study addresses the bacterial decolorization of Indanthrene Blue RS anthraquinone dye and optimizes the parameters required by the bacterial consortium to decolorize the dye effectively. A developed consortium of three organisms, *Bacillus flexus* TS8, *Proteus mirabilis* PMS and *Pseudomonas aeruginosa* NCH, labeled as consortium-BP, was used to decolorize an industrial anthraquinone dye Indanthrene Blue RS and a mixture of various dyes under aerobic conditions. All three bacteria individually evaluated were found to be efficient dye decolorizer. So the consortium of these microorganisms was prepared and utilized for an enhanced decolorization under several optimized physicochemical conditions. A comparative study was conducted with individual strains and the consortium-BP on the enzymatic system and metabolic pathway for Indanthrene Blue RS degradation. The dye degradation was evaluated using UV–vis absorption spectroscopy and FT-IR analysis. The phytotoxicity study was also used to assess the toxicity of the degraded products of Indanthrene Blue RS by the consortium-BP.

## Material and methods

### Dyes and chemicals

Indanthrene Blue RS and other anthraquinone dyes (Vat Green XBN, Vat Brown R, and Vat Yellow 5G) were obtained from Cuttack, Odisha, India. Tartaric acid, n-propanol, ABTS [2,2′-azino-bis(3-ethylbenzothiazoline-6-sulphonic acid)], NADH, methyl red, catechol were purchased from Fisher Scientific, India. Agricultural residues like rice straw and husk, sugarcane bagasse, and wood scrapings were collected from local industries in Odisha, India. These residues were washed and dried and then were milled into a uniform size and stored at room temperature before use. All the chemicals and media components used have been supplied from Hi-media (India) and of analytical grade. The alkaline pH of the media was maintained by using sterilized NaOH (1 M). The textile wastewater sample was collected from local textile processing units, Odisha, India.

### Microorganisms and culture conditions

The pure culture of *Bacillus flexus* TS8, *Proteus mirabilis* PMS, and *Pseudomonas aeruginosa* NCH were isolated from the textile industry effluent containing anthraquinone dyes under laboratory conditions. The culture was grown in a 250 mL flask, each containing 100 mL of nutrient broth, and incubated for 24 h at 30 ºC under aerobic conditions. To analyze the effect of carbon and nitrogen sources on decolorization of Indanthrene Blue RS, liquid mineral-base media (MBM) with composition: 0.3% NaNO_3_, 0.1% K_2_HPO_4_, 0.05% MgSO_4_, 0.05% KCl, 0.02% Yeast Extract, and 0.1% glucose was used. Beef extract, peptone, starch, ammonium sulphate, ammonium chloride, yeast extract, urea, sucrose, glucose, and lactose (1% each) were used as various carbon and nitrogen sources. Rice straw, rice husk, sugarcane bagasse powder, and wood scrapings (1% each) were added individually to 100 mL distilled water and sterilized via autoclave. Aliquots of each agricultural residue (5 mL) were added to the liquid MBM to test for the decolorization efficiency of Indanthrene Blue RS by consortium-BP.

### Development of consortium-BP

One inoculating loop of the individual strains (*Bacillus flexus* TS8, *Proteus mirabilis* PMS and *Pseudomonas aeruginosa* NCH) was added separately into 100 mL nutrient broth each and incubated under aerobic conditions at 30 ºC for 24 h. The consortium-BP was prepared by transferring 50 mL of 24 h grown culture of each strain aseptically into 250 mL flasks to retain the same cell number in the pure culture and consortium, respectively. The consortium-BP was also used as the inoculum in decolorization studies.

### Decolorization experiments

The 24 h consortium-BP was inoculated in a 250 mL flask containing 100 mL of liquid MBM with various textile dyes such as Indanthrene Blue RS, Vat Green XBN, Vat Brown R, and Vat Yellow 5G at a concentration of 100 mg L^−1^. Each flask was inoculated individually (10% (v/v)) and incubated at 30 °C with 150 RPM. Aliquots of 2 mL were withdrawn at regular intervals (1 h). For cell mass separation, each sample was then centrifuged at 8000 RPM for 10 min. Maximum absorption wavelength (λ_max_) was used to check for the reduction in absorbance of each culture supernatant after decolorization for the respective dyes. The individual strains (BF, PM, and PA) and the developed consortium-BP were employed at various initial concentrations of Indanthrene Blue RS (50–250 mg L^−1^) for the decolorization studies at 30 °C, 150 RPM.

Experiments on the effects of pH (7–12) and temperature (30–50 °C) were conducted using 100 mg L^−1^ of Indanthrene Blue RS under aerobic conditions. Further, decolorization studies were conducted by repeatedly adding dye aliquots of 50 mg L^−1^ into the medium without extra nutrients. The developed consortium-BP was used at 100 mg L^−1^ of Indanthrene Blue RS to observe the effects of different carbon and nitrogen sources using the synthetic medium under aerobic conditions. All the decolorization studies were conducted in triplicates. Abiotic controls were included, excluding microorganisms. The decolorization percentage of the dye was deduced as^40^:

1 $$\text{Decolorization }\left({\%}\right)= \frac{(\text{Initial}\; \text{absorbance }-\text{Final} \; \text{absorbance})}{\text{Initial} \; \text{absorbance}} \times 100$$

Average decolorization rate (ADR) (µg h^−1^) has been determined according to the equation^41^:

2 $$\mathrm{ADR}= \frac{(\mathrm{C}\times \mathrm{\%D}\times 1000)}{(100\times \mathrm{t})}$$

where C: initial dye concentration (mg L^−1^), D: dye decolorization percentage (%), t: time after decolorization (h).

### Characterization of real textile effluent

Colour, odor, pH, and temperature were recorded at the site during sampling. In the laboratory, other parameters like electrical conductivity, BOD, COD, TOC, TSS, TDS were determined using standard procedures^42,43^. Heavy metals (chromium, lead, and zinc) were measured using a True Double Beam Atomic Absorption Spectrophotometer SL 176. The colorimetric method was applied to estimate the concentrations of chloride, sulphate, calcium, and sodium.

Consortium-BP was used to determine the COD and the TOC removal ratio before treatment and immediately following the textile effluent's treatment. The reduction in COD was calculated for individual strains and consortium-BP after 9 h of incubation. The COD of the textile effluent was calculated as follows^44,45^:

3 $$\mathrm{COD}\left({\text{mg}\; \text{L}}^{-1}\right)= \frac{8\times \mathrm{C}\times ({\mathrm{V}}_{\mathrm{b}}-{\mathrm{V}}_{\mathrm{a}})}{{\mathrm{V}}_{\mathrm{s}}}$$

where, 8: milliequivalent weight of oxygen, C: titrant concentration, V_a_: titrant volume (control), V_b_: volume of titrant used (sample), Vs: sample volume.

The total organic carbon (TOC) of the textile effluents before and after decolorization was quantified using Shimadzu TOC-VCPN^26^. The residual TOC was assessed for individual strains and consortium-BP before and after 9 h of incubation. The cells were separated by centrifuging at 8000 RPM for 10 min, and the cell-free supernatant was used for TOC analysis, where the measurement was performed at 680 °C with a flow rate of 230 ml min^−1^, carrier gas pressure of about 300 to 600 kPa. The TOC’s removal percentage was deduced as in Eq. (4):

4 $$\text{TOC} \; \text{removal} \; \text{percentage }\left(\mathrm{\%}\right)=\frac{{Initial}_{TOC}-{Final}_{TOC}}{{Initial}_{TOC}} \times 100$$

where initial TOC: values at 0 h, final TOC: values after complete decolorization (9 h), respectively.

### Enzymatic assay

#### Preparation of cell-free extract

The individual strains were grown in 250 mL flasks, each having 100 mL nutrient broth in their respective optimum conditions. The consortium-BP has been developed, as mentioned in “Development of consortium-BP” section. The cultures were then incubated under aerobic conditions for 24 h, followed by centrifugation at 8000 RPM for 15 min to separate the cell mass. The supernatant has been used as an extracellular enzyme source. The biomass acquired from the individual strains and the consortium-BP were then suspended separately before sonication (Qsonica Sonicators) in potassium phosphate buffer (50 mM, pH7.4). The suspensions were then homogenized and sonicated at 4 °C (8 strokes of 40 s each for 2 min interval with the sonifier output kept at 40 amplitude). The supernatant extracted from the sonicated suspension was centrifuged at 8000 RPM for 15 min at 4 °C and utilized as an intracellular enzyme source. Following the decolorization of Indanthrene Blue RS by consortium-BP (complete decolorization) and the individual strains, a similar procedure was followed for assessing enzymatic activities. The Bradford protein assay estimated the crude extract's protein concentration using bovine serum albumin as the protein standard^46^.

#### Oxidative enzymes during decolorization

The oxidative enzymes (lignin peroxidase, laccase, and tyrosinase activity) were evaluated for Indanthrene Blue RS decolorization using spectrophotometry analysis for both intracellular as well as extracellular enzyme source. Substrates such as n-propanol and H_2_O_2_ were used to determine the activity of lignin peroxidase. A 3.0 mL reaction mixture of 100 mM n-propanol, 250 mM tartaric acid was prepared with 10 mM H_2_O_2_. Then the reaction started with the addition of 0.2 mL of enzyme filtrate, equilibrated at 37 °C.

Increased absorbance due to the formation of propanaldehyde was monitored by measuring n-propanol oxidase activity at 300 nm^47^. A reaction mixture of 3.0 mL, comprising of 10% ABTS in 0.1 M acetate buffer at pH 4.9, equilibrated at 37 °C, was assessed for laccase activity. Absorbance increase was monitored at 420 nm^48^. Tyrosinase activity was accessed by modifying the previously reported method using catechol as a substrate in a 2 mL reaction mixture. The reaction mixture equilibrated at 30 °C contained 0.01% catechol in 0.1 M phosphate buffer at pH 7.4^49^. An increase in absorbance was measured at 495 nm. All the enzymatic assays have been done in triplicates, and the enzymatic activities were represented by calculating average rates. One unit of the oxidative enzyme activity was determined as a change in absorbance Units min^−l^ mg of protein^−l^.

#### Reductase enzymes during decolorization

The reductase dye decolorizing enzyme activity such as azoreductase and NADH-DCIP reductase was assessed by following the method found by Karim et al.^50^ The assay was conducted in a 2.0 mL reaction mixture that included 100 µM NADH and 4.45 µM of Methyl red in 50 mM phosphate buffer (pH 7.4) with 0.1 mL enzyme filtrate. The reaction mixture was equilibrated at room temperature for 4 min and initiated by adding NADH. The decrease in optical density was monitored at 430 nm. A significant reduction in methyl red was determined by using a molar extinction coefficient of 0.023 µM^−1^ cm^−1^.

The NADH-DCIP reductase activity was recorded by modifying the reported procedure^51^. The reaction mixture of 5.0 mL contained 50-lM DCIP, 50-lM NADH in 50 mM potassium phosphate buffer (pH 7.4) with 0.1 ml of enzyme filtrate and was assayed at 595 nm. The reduction of DCIP was measured by using the molar extinction coefficient of 90 mM^−1^ cm^−1^. All the enzymatic assays were conducted in triplicates. One unit of the reductase activity was established by the quantity of enzyme needed to reduce 1 µM of substrate min^−l^ mg of protein^−l^.

### Biodegradation studies

Decolorization of Indanthrene Blue RS was observed in the visible range of 400–700 nm with Shimadzu UV-1800. During the decolorization study, 2 mL aliquots were withdrawn at zero hours and later on at a regular time interval (1 h). The cells were collected by centrifuging the sample at 8000 RPM for 10 min. The biodegradation was monitored and confirmed by FT-IR spectroscopy. FT-IR analysis was conducted to evaluate the modifications in the functional groups of the extracted metabolites before and after decolorization. After complete decolorization, the supernatant was collected by centrifuging 50 mL of the culture medium at 8000 rpm for 10 min. The degraded dye products were then extracted using ethyl acetate in 1:1 proportion and dried using a rotary evaporator. The residual deposits were dispersed in HPLC grade methanol until further study. FT-IR analysis was performed using Thermofisher Scientific, Nicolet IS10 in ATR mode with a 16-scan speed of 400–4000 cm^−1^ in the mid-IR range.

The GC–MS analysis was performed to determine the compounds involved in the metabolic pathway of Indanthrene Blue RS degradation towards mineralization by consortium-BP. The study was used to identify the metabolites formed during each stage of Indanthrene Blue RS dye degradation. Agilent 7890-B Gas Chromatography and 5977-A Mass Spectrometry were used to analyze the degraded metabolites. The experiment was carried out in temperature programming mode with a 70 eV ionization voltage. The splitless mode of the front inlet was maintained. The helium gas flow rate was maintained at 1.0 ml min^−1^. The initial oven temperature was held at 80 °C for 1 min, then increased to 210 °C at a rate of 12 °C min^−1^. The temperature was then raised to 230 °C at a rate of 15 °C min^−1^ and maintained at that temperature for 4 min. Afterward, the temperature was increased to 250 °C at 3 °C min^−1^, and then to 300 °C at 40 °C min^−1^ subsequently. The highest resolution chromatographic peaks were screened for mass fragmentation and were characterized based on their mass spectrum similarities to those in the NIST library.

### Phytotoxicity studies

Untreated textile effluents on discharge cause pollution and discoloration of water bodies, inhibiting photosynthesis and affecting the growth of aquatic life^52^. After degradation, the toxic effects of the dye and its metabolites were evaluated with 500 mg L^−1^ of Indanthrene Blue RS through phytotoxicity tests, with two types of seed frequently used in agriculture: *Triticum aestivum* and *Phaseolus mungo*. Studies were carried out using 100 mm plastic Petri dishes with two layers of sterilized Whatman filter paper. Ten seeds from each crop were grown separately in the dark with the control dye, and metabolites were extracted. Seeds provided with distilled water were used as a control. Toxicity studies were carried out at 30 °C and the same optimal conditions by providing an equal volume of distilled water, dye, and the extracted metabolites (10 mL per day) to the seeds. The experiments were performed in triplicates. After 7 days, the toxicity effects were recorded from the seedlings' growth by measuring the plumule and radical length, germination (%).

5 $$Germination \left(\%\right)= \frac{No. \; of \;seeds \; germinated}{No. \; of \; seeds \; sowed} \times 100$$

### Statistical analysis

One-way analysis of variance (ANOVA) and Tukey’s-HSD comparison studies was carried out using IBM SPSS statistics 23.0 to analyze the observed data (Enzyme and Phytotoxicity analysis). The readings were considered to be significant, with a *P*-value ≤ 0.05.

## Results and discussion

### Comparative studies for the decolorization of various reactive dyes

Previous studies have shown that bacterial cells are a cost-effective and promising method for degrading different dyes found in textile effluents. A microorganism’s potential to degrade a dye can be evaluated by its decolorization capacity^24,53,54^. The microbial consortium generates enzymes that are highly effective in the decolorization of the dyes due to their improved survival, adaptability, and enzymatic activity. In this study, the ability of both the developed consortia and the pure culture to decolorize various anthraquinone dyes viz. Indanthrene Blue RS, Vat Green XBN, Vat Brown R, and Vat Yellow 5G have been assessed. Decolorization was performed in the liquid-mineral broth with dye supplementation (100 mg L^−1^ each) under aerobic conditions.

Table 1 illustrates the potential of consortium-BP in the decolorization of different anthraquinone dyes with a higher ADR relative to the individual strains. The results showed that consortium-BP could completely decolorize Indanthrene Blue RS within 9 h with a maximum ADR of 11,088 µg h^−1^, compared to that of *B. flexus* (4083 µg h^−1^) in 24 h, *P. mirabilis* (3833 µg h^−1^) in 20 h, and *P. aeruginosa* (3708 µg h^−1^) in 14 h. The higher ADR of the consortium-BP could be attributed to the synergistic response of the individual bacterial isolate in the consortium-BP^55^. As a result, we concluded that for complete decolorization of the dye, *B. flexus*, *P. mirabilis,* and *P. aeruginosa* required more time. The slow rate of decolorization by individual strains can be related to higher molecular weights and the structural differences in the dyes^18,56^.

**Table 1:** Average decolorization rate of individual strains and the consortium-BP of various anthraquinone dyes (100 mg L^−1^).

Name of anthraquinone dyes	λ_max_ (nm)	Average decolorization rate (µg h^−1^)			
		BF	PM	PA	BP
Indanthrene Blue RS	520	4083	3833	3708	11,088
Vat Green XBN	550	2450	4750	3100	9222
Vat Brown R	610	1388	1800	4944	4667
Vat Yellow 5G	580	933	566	1266	2556

*BF Bacillus flexus* TS8; *PM Proteus mirabilis* PMS; *PA Pseudomonas aeruginosa* NCH; *BP* developed consortium.

### Effect of physicochemical parameters on decolorization

Microbial decolorization of recalcitrant Indanthrene Blue RS dye was investigated by studying the effects of different physicochemical variables such as pH, temperature, dye concentration, and agitation with individual pure cultures (BF, PM, and PA) as well as consortium-BP. The experiment was conducted with 100 mg L^−1^ of Indanthrene Blue RS dye at temperatures ranging from 30 to 50 °C. The consortium-BP revealed faster decolorization within 9 h than the Individual strains (PA, PM, and BF) within 14, 20, 24 h of incubation and at an optimum temperature of 35 °C, respectively. As the temperature increases, there was a rapid decrease in degradation efficacy for both individual strains and consortium-BP. The transfer of dye molecules to the cell depends on the pH. It is assumed to become a rate-limiting phase for the decolorization of the dye^57^, making it necessary to assess the effect of pH on decolorization. Individual strains (BF, PM, and PA) and consortium-BP showed maximum decolorization activity for Indanthrene Blue RS at pH 10 (Table 2). There was a decrease in decolorization efficacy at acidic pH in all three cultures. Consortium JW-2, as stated by Moosvi et al. reflected a similar outcome^56^.

**Table 2:** Decolorization performance and an incubation time of Indanthrene Blue RS using individual strains and developed consortium-BP at different temperatures, pH, and dye concentrations.

Strain	Parameters	Operational conditions															
		Temperature (ºC)						pH					Dye concentration (mg L^−1^)				
		30	35	37	40	45	50	8	9	10	11	12	50	100	150	200	250
BF	Decolorization (%)	CD	CD	92	80	42	28	48	72	CD	64	36	CD	CD	90	50	28
	ADR (mg h^−1^)	4.85	4.08	3.83	2.22	0.88	0.38	1.2	3.6	4.2	2.13	0.75	5.0	4.16	3.0	1.38	0.58
	Time (h)	24	20	24	36	48	72	40	20	24	30	48	20	24	30	36	48
PM	Decolorization (%)	89	CD	CD	53	40	32	52	60	CD	51	ND	CD	CD	82	48	23
	ADR (mg h^−1^)	2.47	3.57	5.0	1.1	0.55	0.44	1.44	2.5	5.0	1.06	–	5.55	5.0	2027	0.67	0.32
	Time (h)	36	28	20	48	72	72	36	24	20	48	72	18	20	36	72	72
PA	Decolorization (%)	CD	CD	CD	65	59	42	57	68	CD	42	ND	CD	CD	CD	58	40
	ADR (mg h^−1^)	5.55	5.0	4.17	1.35	1.22	0.58	1.9	2.83	5.55	0.87	–	5.0	5.55	4.17	1.21	0.55
	Time (h)	18	20	24	48	48	72	30	24	18	48	72	20	18	24	48	72
BP	Decolorization (%)	CD	CD	94	85	78	62	68	80	CD	76	52	CD	CD	CD	96	CD
	ADR (mg h^−1^)	8.3	11.1	3.92	2.36	1.63	0.86	1.42	6.66	11.1	3.17	1.44	8.3	11.1	4.17	3.2	4.17
	Time (h)	12	09	24	36	48	72	48	12	09	24	36	12	09	24	30	24

ADR: Average decolorization rate (mg h^−1^), CD: complete decolorization, ND: no decolorization, *BF: Bacillus flexus* TS8, *PM: Proteus mirabilis* PMS, *PA: Pseudomonas aeruginosa* NCH, *BP:* developed consortium.

Table 2 illustrates the decolorization activity of individual strains and consortium-BP over varied Indanthrene Blue RS concentration (50, 100, 150, 200, and 250 mg L^−1^). Decolorization with dye concentrations of 50, 100, and 150 mg L^−1^ resulted in complete decolorization by the individual strains and consortium-BP. In comparison, a decreased decolorization efficacy was observed with a higher dye concentration of 200 and 250 mg L^−1^, respectively. This significant reduction in the decolorization efficiency of the dye at high concentrations can be attributed to the toxic effect of the dye on the bacteria in the consortium or due to insufficient biomass concentration needed to absorb higher concentrations of dye^57,58^. The consortium-BP showed faster decolorization efficiency at high dye concentrations of 200 and 250 mg L^−1^ of Indanthrene Blue RS (Table 2). Textile wastewaters usually contain dye at a concentration of about 16–32 mg L^−1^^59^, making the consortium-BP a better candidate for dye degradation. It could fit well into a continuous process in the treatment of textile effluent containing different anthraquinone dyes.

### Effect of co-substrate addition on decolorization

Dyes are deficient sources of carbon. For biodegradation studies using a bacterial consortium, it is essential to include a carbon and nitrogen source to the dye^60^. Efficient decolorization of Indanthrene Blue RS by consortium-BP was studied with the addition of carbon and nitrogen-containing sources (1%) and extracts (5 mL) from each agricultural residue to the media. Maximum decolorization efficacy was observed with substrate yeast extract and glucose (100%) within 9 h. In contrast, with 5 mL extracts of agricultural residues, i.e., rice husk and wood scrapings, complete decolorization was observed within 24 h of incubation. Jadhav et al. reported an increase in Direct Blue GLL decolorization by supplementing agricultural waste extracts resulting in the production of ligninolytic enzymes by *Comamonas* sp UVS^52^. Decolorization of PolyR-478 has been reported in *Phanerochaete chrysosporium* culture, where the concentration of grape seeds and wood shavings have been used as additives^60^.

The consortium-BP exhibited very low to moderate decolorization within 24 h by Ammonium chloride (18%), Lactose (22%), Peptone (25%), Yeast extract + Lactose (32%), Sucrose (39%), Yeast extract + Sucrose (43%), Beef extract (47%), glucose (67%), and Yeast extract (73%). With the extract of rice straw and sugarcane bagasse, consortium-BP showed moderate decolorization of 76% and 37%, respectively, within 24 h of incubation. No decolorization was observed for synthetic media (MBM), ammonium sulfate, starch, and urea.

**Figure 1:**
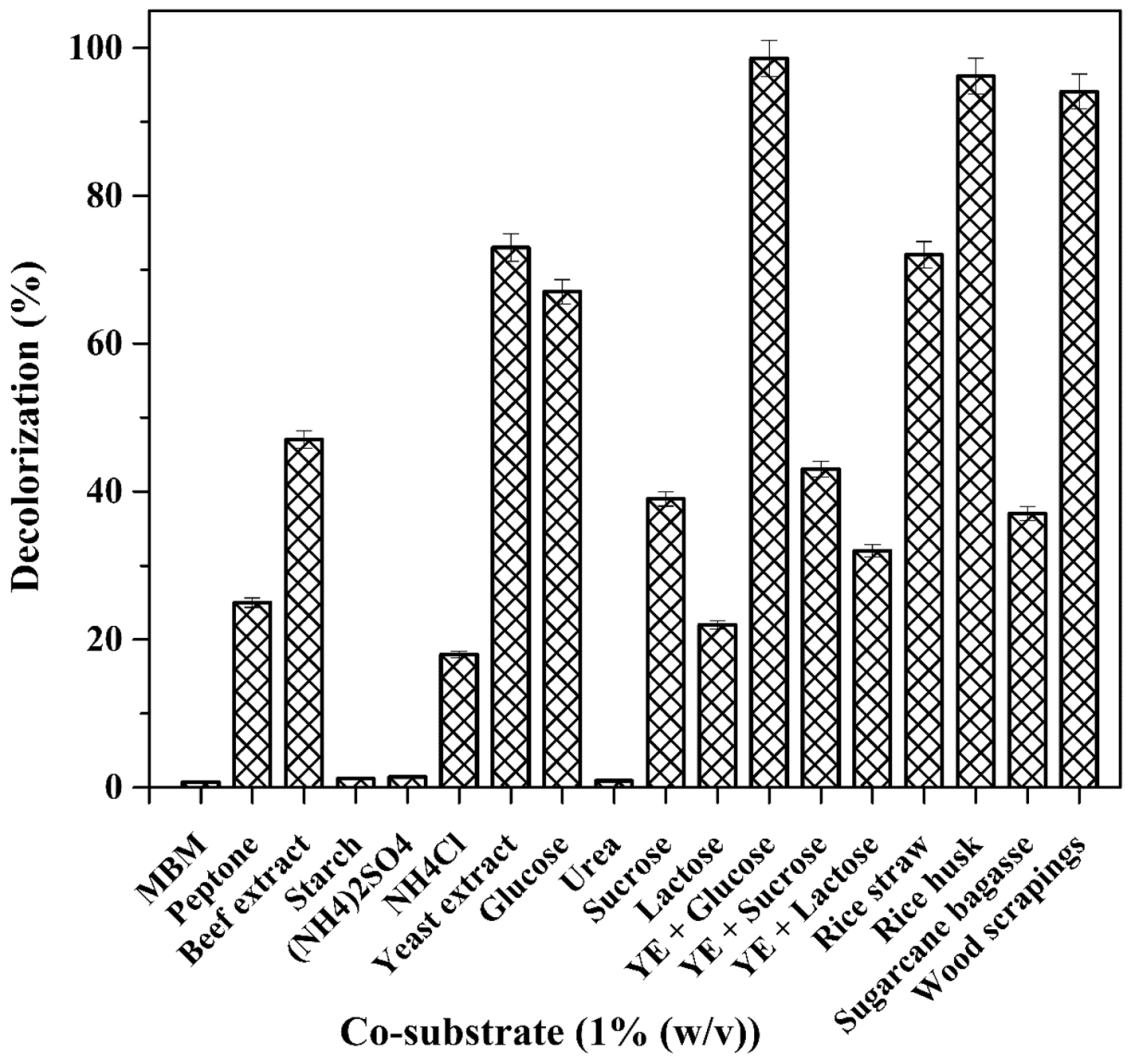
Effect of supplementation of different co-substrate on the Indanthrene Blue RS decolorization by consortium-BP.

All the above findings concluded that a higher decolorization efficiency of Indanthrene Blue RS was achieved with consortium-BP when carbon and nitrogen source (yeast extract + glucose, 5 mL extracts of rice husk and wood scrapings) were used as supplements (Fig. 1). Jadhav et al. demonstrated that using carbon and nitrogen sources as additives can have a stimulating or inhibitory effect on the dye degrading enzyme systems^52^. Consortium-BP is supposed to transform and degrade substrates like rice husk and rice straw to produce volatile organic compounds (acetic acid) and alcohols (ethanol). These nitrogen sources can produce NADH that acts as electron donors, thus inducing microorganisms to reduce the azo dyes^51,52^. The use of agricultural residues like rice husks and wood scrapings as a substrate can increase the decolorization of Indanthrene Blue RS dye is more cost-effective. It also solves the issue of agro-residues disposal. Besides being eco-friendly, it is also inexpensive, so it can be used instead of existing physicochemical methods to degrade industrial effluents^45^.

### Decolorization with repeated dye addition

**Figure 2:**
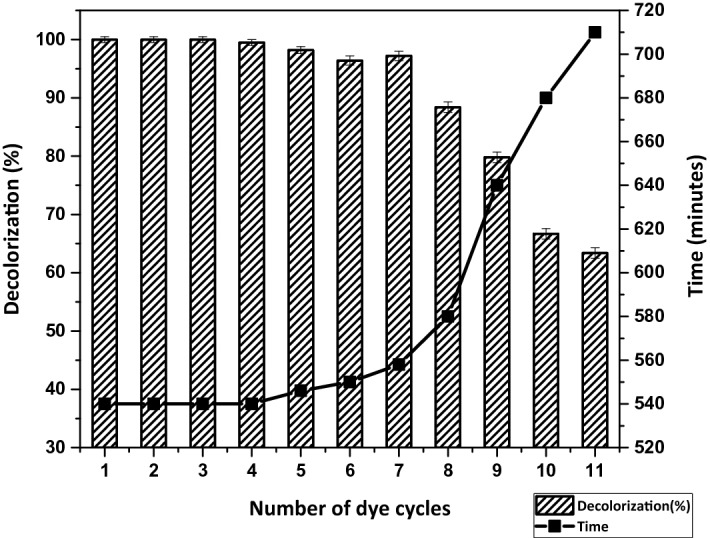
Effect of repeated dye addition on decolorization.

The repeated use of cell biomass for industrial purposes is an economic parameter in the process of decolorization. The experiment was carried out under optimal conditions to evaluate the ability of individual strains (BF, PM, and PA) and consortium-BP to repeatedly decolorize Indanthrene Blue RS dye (100 mg L^−1^). Individual strains can decolorize up to 3rd repeated dye cycles. The consortium-BP can maintain up to the 7th cycles with a slight difference in percentage decolorization and time requirements, thus increasing the consortium's relevance in decolorizing real wastewater. Figure 2 illustrates a decrease in decolorization efficiency from the 6th to the 9th cycle, increasing the time requirements. The microbial culture entering the stationary phase and successively into the death phase may result in a gradual reduction in decolorization, resulting in the inhibition of enzyme systems. Similarly, no decolorization was observed in the 11th dye cycle. The exhaustion of the nutrients in the medium may result in the decolorization process being completed^61^.

### Biodecolorization of textile effluent

Supplementary Table 2 illustrates the physicochemical parameters of the collected effluent before and after treatment. The water sample collected was dark grey and had a pungent fishy odor due to the higher concentration of dyes. The alkaline pH was attributed to the use of a large amount of salt during the dyeing process. The high temperature was attributed to hot water use during the vatting process, typically decreasing the level of dissolved oxygen in the water. The higher concentration of ions and dyes in water resulted in increased electrical conductivity. Higher BOD suggested a reduction in oxygen level and the toxic nature of water. Simultaneously, the increased COD showed the use of an excessive amount of detergents and dye-fixing agents, thereby increasing the toxicity and the accumulation of biologically resistant organic compounds in the water system. The high TDS value is mainly due to the fixing, bleaching, and dyeing agents used at various fabric processing stages. The high TDS value of water is not recommended for consumption and agricultural uses because it can cause salinity problems. The higher TSS value is attributed to increased suspended particles in the effluent, showing increased water bodies' turbidity. The oxygen level in the water system is reduced, thereby disrupting the food chain in the aquatic environment. Moreover, the higher values of the untreated sample in the textile effluent get reduced after its treatment. In the present study, chloride, sulfate, calcium, and heavy metals contents (chromium, zinc, and lead) in textile effluents are lower than the BIS permissible limits.

**Figure 3:**
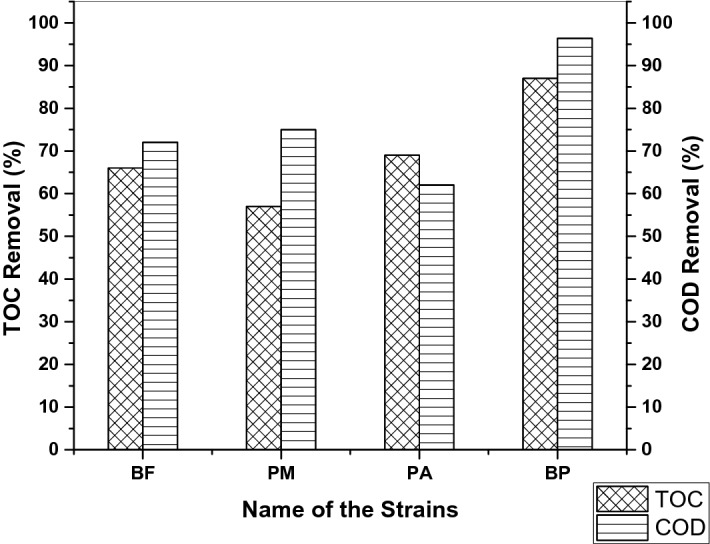
TOC and COD removal percentage of Indanthrene Blue RS by individual strains and consortium-BP after 9 h incubation.

In the textile processing industry, various anthraquinone dyes with fused aromatic rings are commonly used and, thus, differ in the composition of their waste stream. In this study, consortium-BP decolorized Indanthrene Blue RS by 99% and textile effluent by 82% within 9 h of incubation. Individual strains can decolorize Indanthrene Blue RS dye efficiently within 24 h. Selvakumar et al. reported a higher decolorization efficiency with the bacterial consortium for several textile dyes^32^. The intermediates formed during decolorization and biodegradation of anthraquinone dyes may be prolonged and highly toxic than that of the parent compound. Mineralization percentage, i.e., TOC and COD removal percentage, were determined by individual strains and consortium-BP from the decolorization of Indanthrene Blue RS. Consortium-BP showed a decreasing TOC (87%) and COD (96.4%) removal ratio by completely decolorizing Indanthrene Blue RS within 9 h, which is much higher than the individual strains (Fig. 3). The substantial removal of TOC and COD levels shows that the toxic substances of the effluent are reduced. The increased decolorization rate with the bacterial consortium may be attributed to the synergism between the enzymatic system and surface area accessibility.

### Enzyme analysis

Microorganisms use their versatile enzyme systems to decolorize the dyes. Induction of decolorization mechanism by lignin peroxidase, laccase, tyrosinase, NADH–DCIP reductase, and azoreductase were observed in this study after 9 h incubation with consortium-BP, *B. flexus*, *P. mirabilis*, and *P. aeruginosa,* respectively. Significant induction of enzymatic activity was observed in intracellular lignin peroxidase (375%), extracellular lignin peroxidase (332%), laccase (311%), tyrosinase (350%), and azoreductase (507%) by consortium-BP culture compared with individual strains of *B. flexus*, *P. mirabilis*, and *P. aeruginosa* respectively. Similarly, moderate inductions by consortium-BP were detected in laccase (267%), NADH-DCIP reductase (239%), tyrosinase (233%), and intracellular lignin peroxidase (227%) activity relative to *B. flexus* (Table 3).

**Table 3:** Activities of the dye degrading enzymes after 9 h in consortium-BP (complete decolorization), *Bacillus flexus* TS8, *Proteus mirabilis* PMS, and *Pseudomonas aeruginosa* NCH.

Enzymes	Df	F-value	P-value	*Bacillus flexus*	*Proteus mirabilis*	*Pseudomonas aeruginosa*	Consortium-BP
Lignin peroxidase (intracellular)^a^	11	30.339	0.000*	0.849 ± 0.03	1.023 ± 0.03	0.514 ± 0.37	1.925* ± 0.012
Lignin peroxidase (extracellular)^a^	11	20.324	0.000*	0.072 ± 0.023	0.113 ± 0.029	0.153* ± 0.040	0.239** ± 0.000
Laccase^a^	11	7.113	0.012*	0.021 ± 0.01	0.018 ± 0.009	0.036 ± 0.008	0.056* ± 0.004
Tyrosinase^a^	11	11.685	0.003*	0.015 ± 0.006	0.010* ± 0.005	0.022 ± 0.005	0.035** ± 0.004
NADH-DCIP reductase^b^	11	18.538	0.001*	11.84 ± 3.36	16.29* ± 4.07	20.84 ± 1.60	28.34*** ± 1.26
Azoreductase^c^	11	60.456	0.000*	1.97 ± 0.74	4.75 ± 0.56	6.63*** ± 0.91	9.98*** ± 0.75

df: degrees of freedom, Values are mean of three experiments, SEM ( ±), significantly different from *Bacillus flexus* at *P < 0.05, **P < 0.01, and ***P < 0.001 and by one-way ANOVA with Tukey’s-HSD Comparisons Test.

^a^U min^−1^ mg protein^−1^.

^b^µg of DCIP reduced min^−1^ mg protein^−1^.

^c^µM of methyl red reduced min^−1^ mg protein^−1^.

In contrast, consortium-BP slightly induced intracellular lignin peroxidase (188%), NADH-DCIP reductase (174%), and azoreductase (151%) compared to *P. mirabilis*. Likewise, a moderate induction of azoreductase (210%) and laccase (212%) whereas slight induction of extracellular lignin peroxidase and laccase (156%), tyrosinase (159%), NADH-DCIP reductase (136%) activity by consortium-BP relative to *P. aeruginosa* respectively was demonstrated as illustrated in Table 3. The synergistic effect of three microorganisms may result in a higher induction of oxidoreductive enzymes in consortium-BP. This enzymatic mechanism causes the consortium-BP to decolorize Indanthrene Blue RS in less time than individual strains.

Bradford method has estimated the protein concentration of the isolated intracellular and extracellular enzymes. The higher protein concentration of around 286 mg mL^−1^ was observed in the intracellular enzyme, while a low protein concentration of about 62 mg mL^−1^ was found in the extracellular enzyme (Supplementary Table 1).

### Biodegradation analysis

Supplementary Figure 1 illustrates changes in Indanthrene Blue RS absorbance peaks during biodegradation. UV–vis spectral analysis (400–800 nm) of Indanthrene Blue RS exhibits a λ_max_ (maximum absorbance) of 520 nm. A gradual decrease in both the concentration of dye and absorbance indicates the removal of the dye. Thus, decreased absorbance of Indanthrene Blue RS revealed the rapid degradation of the dye. The disappearance of the significant absorbance peak and the pellet's color leads to the conclusion that the decolorization of Indanthrene Blue RS by consortium-BP might be due to biodegradation associated with bioabsorption^20^.

The FT-IR spectrum analysis of the control dye and the extracted metabolites indicate the biodegradation of the parent dye compounds by the consortium-BP (Supplementary Figure 2). The peaks in the control dye spectrum suggest O–H bonding at 1395 cm^−1^, >C=O stretching groups at 1730 cm^−1^, C–H stretching group at 2970 cm^−1^, and –NH stretching vibration at 3350 cm^−1^, respectively. The FT-IR spectrum of the extracted metabolites demonstrates a long narrow peak around 3350 cm^−1^ due to the –NH stretching vibration of aromatic amines coupled with the –OH stretching group. Another relatively large peak near 1730 cm^−1^ is attributed to the ketone >C=O stretching groups. From the FT-IR spectrum, we can infer that the molecule has split along the N–H bonds and has resulted in amine bond formation.

**Figure 4:**
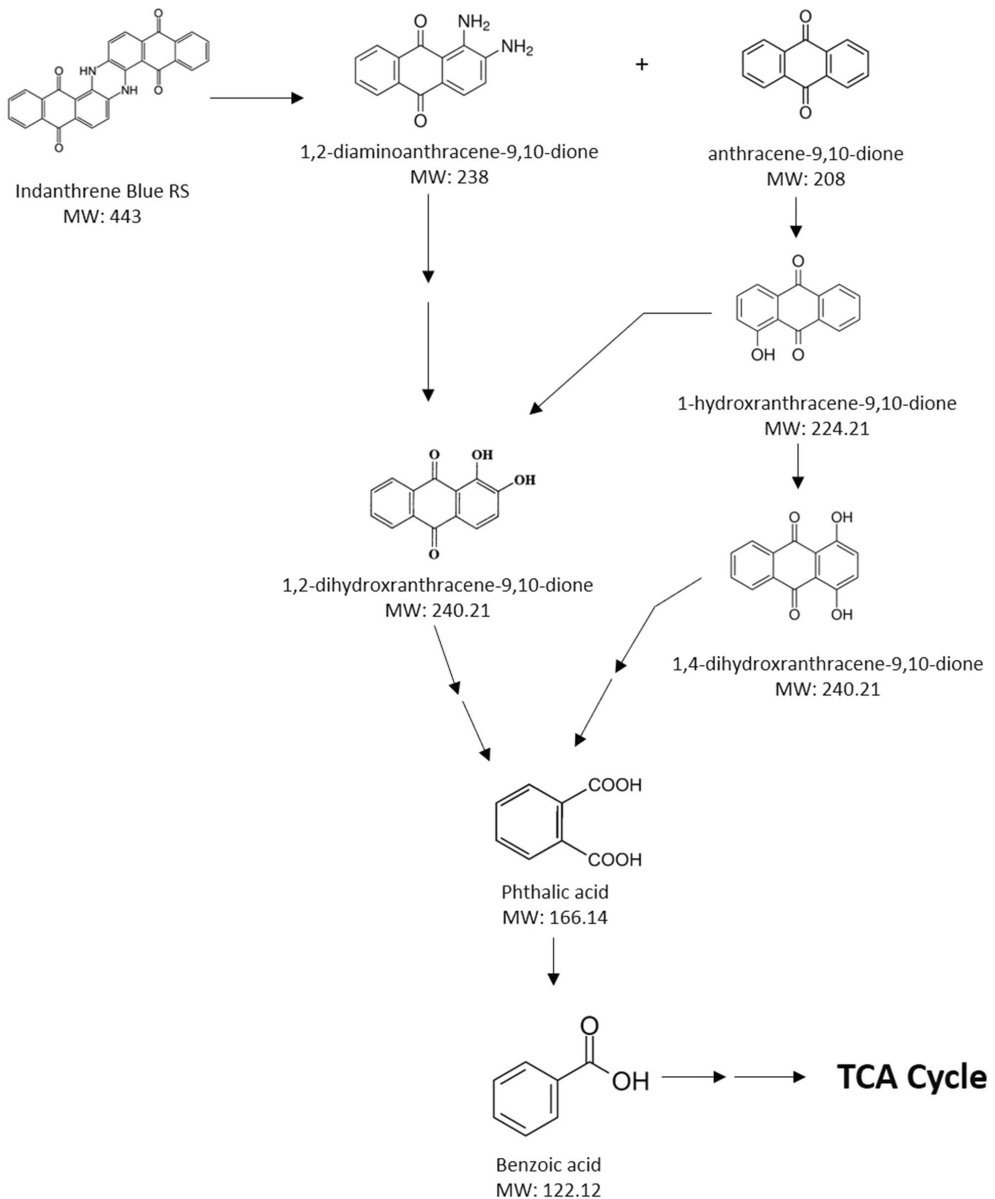
The proposed metabolic pathway of Indanthrene Blue RS degradation towards mineralization by consortium-BP.

The GC–MS analysis identified the metabolic pathway of Indanthrene Blue RS degradation towards mineralization by consortium-BP (Table 4). The production of oxidoreductase enzymes by consortium-BP results in Indanthrene Blue RS dye fragmentation at –NH bond. Indanthrene Blue RS dye degradation resulted in two different ways, were identified as 1,2-diaminoanthracene-9,10-dione (m/z: 238) and anthracene-9,10-dione (m/z: 208). The former one (1,2-diaminoanthracene-9,10-dione) after deamination fragmented into 1,2-dihydroxyanthracene-9,10-dione (m/z: 240.2), which after dehydroxylation converts into phthalic acid (m/z: 166.1). The other one (anthracene-9,10-dione) after dehydroxylation converted into 1-hydroxyanthracene-9,10-dione (m/z: 224.2), 1,4-dihydroxyanthracene-9,10-dione (m/z: 240.2), and phthalic acid (m/z: 166.1). The phthalic acid (m/z: 166.1) further undergo dehydroxylation and converts benzoic acid (m/z: 122.1). The hydroxyl radical (OH·) has been shown to be an effective oxidant for reducing a variety of organic compounds^62^. The action of hydroxyl radical (OH·) might be responsible for the mineralization of Indanthrene Blue RS dye by consortium-BP. Benzoic acid undergoes ring fission and successive transformations, resulting in TCA cycle intermediates in microorganisms^57,58^. The formation of benzoic acid as an end-product suggested converting toxic compounds via oxidative metabolic process to a non-toxic compound. The detailed proposed pathway of Indanthrene Blue RS degradation towards mineralization by consortium-BP is illustrated in Fig. 4.

**Table 4:**
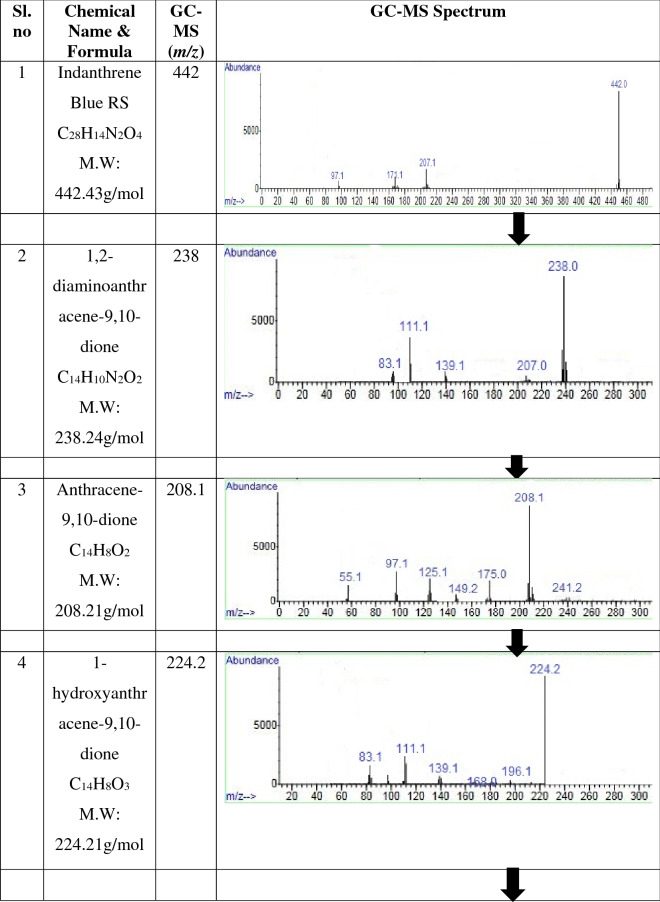

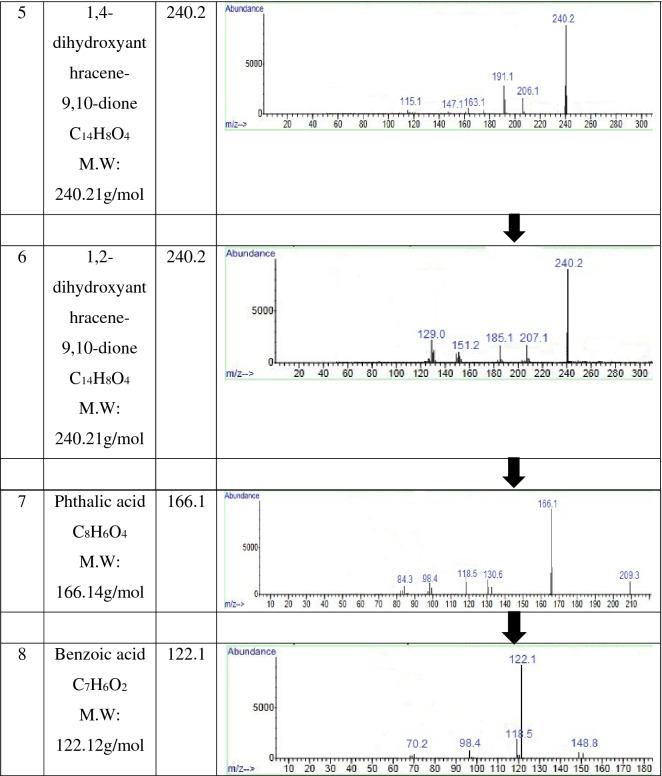
Compounds involved in the metabolic pathway of Indanthrene Blue RS degradation towards mineralization by consortium-BP, identified by GC–MS analysis.

### Phytotoxicity analysis

This study demonstrated that there is an inhibitory effect on the growth of the seedlings containing 500 mg L^−1^ of Indanthrene Blue RS both for *T. aestivum* and *P. Mungo* by 40 and 60%, respectively. In contrast, the inhibitory effect with real textile effluent was 50 and 60%, respectively (Table 5). Significant growth was observed in the length of the plumule and radicle and complete germination of seedlings containing metabolites of degradation by the dye. Similar results have been seen in other studies by Telke et al., and Jadhav et al.^41,60^.

**Table 5:** Phytotoxicity studies of Indanthrene Blue RS, textile effluent, and its metabolites formed after degradation by consortium-BP.

Parameters	df	f-value	p-value	*T.aestivum*			df	f-value	p-value	*P. mungo*		
				Distilled water	Indanthrene Blue RS^a^	Extracted metabolites^a^				Distilled water	Indanthrene Blue RS^a^	Extracted metabolites^a^
Germination (%)				100	60	100				100	40	100
Plumule (cm)	8	9.311	0.014*	5.07 ± 0.19	3.37 ± 0.65	4.30 ± 0.49*	8	35.666	0.000*	8.16 ± 0.86	2.20* ± 0.43	6.34 ± 1.18
Radicle (cm)	8	17.397	0.003*	2.64 ± 0.75	0.75 ± 0.48*	3.28 ± 0.31	8	50.393	0.000*	2.22 ± 0.29	0.56** ± 0.08	2.35 ± 0.29

df: degrees of freedom.

Values are mean of three experiments, SEM ( ±), significantly different from the seeds germinated in distilled water (control) at *P < 0.05, **P < 0.01, by one-way ANOVA with Tukey’s-HSD Comparisons Test.

^a^Concentration = 500 mg L^−1^.

We, therefore, conclude from this study that the textile effluents containing Indanthrene Blue RS dye treated with consortium cultures resulted in the complete degradation of the dye with the treated effluent being used for agri-irrigation that indicates the nontoxic nature of metabolite products.

## Conclusion

Developed bacterial consortium-BP, which included *Bacillus flexus* TS8, *Proteus mirabilis* PMS and *Pseudomonas aeruginosa* NCH, effectively decolorizes and degrades Indanthrene Blue RS dye under aerobic conditions. With a significant decolorization rate, the consortium was able to degrade Indanthrene Blue RS and required less incubation time compared with individual strains. Agricultural waste extracts such as rice husk and wood scrapings have been identified as better supplements to increase Indanthrene Blue RS decolorization by consortium-BP, making it more cost-effective. Significant induction of oxidoreductive enzymes in consortium-BP indicates the change in metabolism through absorption of Indanthrene Blue RS compared to that of Individual strains. The consortium-BP showed a significant reduction in the physicochemical parameters of the textile wastewater within the permissible limit. Thus, the quality of the treated wastewater can be recommended for irrigation in agricultural fields. The study of TOC and COD removal revealed that this dye could be completely mineralized by consortium-BP with non-toxic metabolites assessed through phytotoxicity studies. Biodegradation analysis showed the detoxification of the parent compound with consortium-BP and individual strains forming metabolites. Benzoic acid was the end-product of the proposed degradation pathway for the Indanthrene Blue RS dye, as confirmed by GC–MS analysis. The mineralized metabolite shows the existence of oxidoreductase enzymes into non-toxic compounds. The above results suggest that the developed consortium-BP could be used as a promising method for practical applications in decolorizing and simultaneously minimizing the toxicity of textile effluents containing anthraquinone dyes. The information gained from the biodegradation process and the enzymatic mechanisms involved in the degradation of reactive dyes provide better knowledge of the transformation of anthraquinone-based dyes in the environment and solve the critical challenges at the industrial level.

## Supplementary Information

Supplementary Information.

## Data Availability

Data are however available from the authors upon reasonable request and with permission.
