# Supplementary material for: Enhanced degradation of anthraquinone dyes by microbial monoculture and developed consortium through the production of specific enzymes
Source: Sci Rep. 2021 Apr 7;11:7678. doi: 10.1038/s41598-021-87227-6 (PMC8027401; doi:10.1038/s41598-021-87227-6)
Supplement: Supplementary file 1 — Supplementary Information. [file 41598_2021_87227_MOESM1_ESM.docx]

**Enhanced degradation of anthraquinone dyes by microbial monoculture and developed consortium through production of specific enzymes**

**Swati Sambita Mohanty^*^, Arvind Kumar**

Department of Chemical Engineering, National Institute of Technology Rourkela, Rourkela, Odisha 769008, India.

*Corresponding Author: Email: 514CH1007@nitrkl.ac.in, Tel: +917205731730.

**Supplementary Table 1**. Protein estimation in enzymes

| **Sample** | **Protein concentration (mg mL^-1^)** |
| --- | --- |
| Intracellular enzyme protein | 286 |
| Extracellular enzyme protein | 62 |

**Supplementary Table 2.** Physico-chemical properties of textile wastewater

| **Parameters** | **Untreated sample** | **BIS (Bureau**  **of Indian**  **Standards) limits** | **Treated**  **sample** | **%**  **Reduction** |
| --- | --- | --- | --- | --- |
| Color (Indanthrene Blue RS)  (optical density value) | Dark grey  1.58 | Colorless | Creamish white  0.003 | 99.8 |
| Odor | Pungent fishy smell | Odorless | Odorless | - |
| pH | 9.6 | 5.5-8 | 7.2 | - |
| Temperature ^0^C | 45 | 40 | 35 | - |
| Electrical Conductivity (µmho cm^-1^) | 2825 | 2500 | 2386 | - |
| BOD, mg L^-1^ | 87.6 | 30 | 6.24 | 92.8 |
| COD, mg L^-1^ | 453 | 250 | 16.31 | 96.4 |
| TOC, mg L^-1^ | 1345 | Up to 1000 | 174.85 | 87 |
| TDS, mg L^-1^ | 2634 | 2100 | 456.4 | 82.6 |
| TSS, mg L^-1^ | 534 | 100 | 63.2 | 88.2 |
| Chlorides, mg L^-1^ | 128.4 | 1000 | - | - |
| Sulphate, mg L^-1^ | 156 | 400 | - | - |
| Calcium, mg L^-1^ | 69.3 | 200 | - | - |
| Chromium, mg L^-1^ | 1.72 | 2 | - | - |
| Lead, mg L^-1^ | 0.006 | 0.1 | - | - |
| Zinc, mg L^-1^ | 3.96 | 15 | - | - |

**Supplementary Figure 1**. UV-vis spectrum of Indanthrene Blue RS obtained after degradation by using *Bacillus flexus* (BF)*, Proteus mirabilis* (PM)*, Pseudomonas aeruginosa* (PS), and consortium-BP.


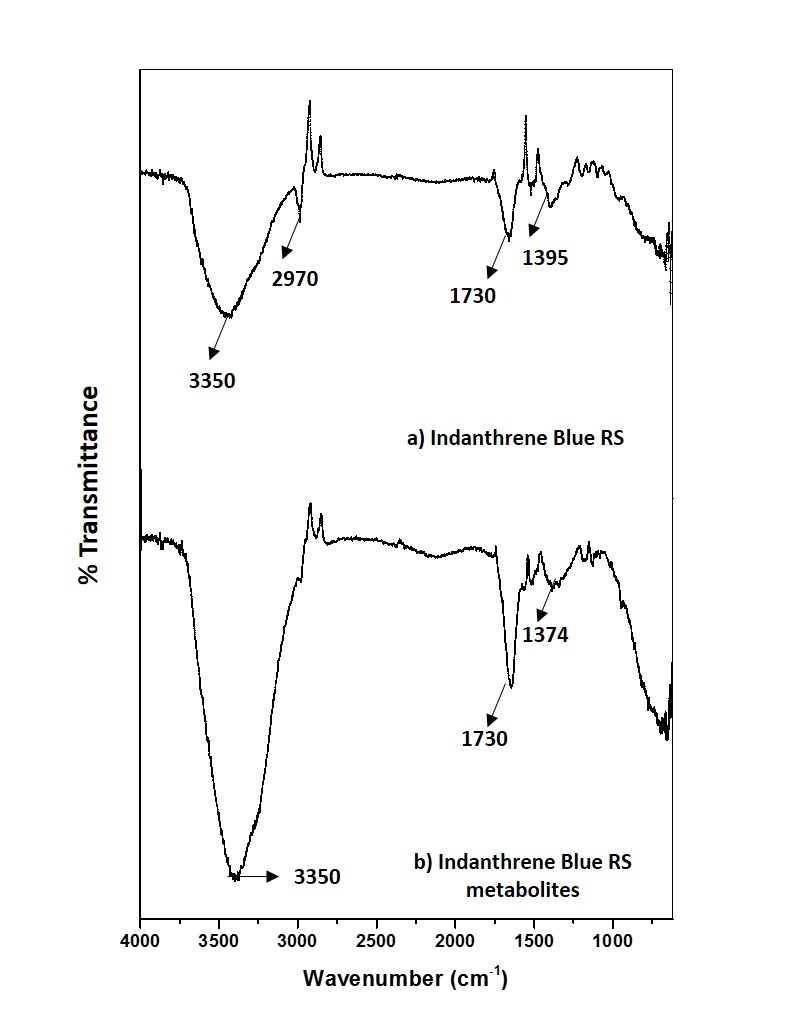


**Supplementary Figure 2.** FTIR spectrum of control dye Indanthrene Blue RS and its metabolites obtained after degradation by using consortium-BP.
